# Effectiveness of Repetitive Transcranial Magnetic Stimulation in the Treatment of Bipolar Disorder in Comparison to the Treatment of Unipolar Depression in a Naturalistic Setting

**DOI:** 10.3390/brainsci12030298

**Published:** 2022-02-23

**Authors:** Abdullah Alhelali, Eisa Almheiri, Mohamed Abdelnaim, Franziska C. Weber, Berthold Langguth, Martin Schecklmann, Tobias Hebel

**Affiliations:** Department of Psychiatry and Psychotherapy, University of Regensburg, 93053 Regensburg, Germany; abdullah.alhelali@medbo.de (A.A.); eisa.almheiri@medbo.de (E.A.); mohamed.abdelnaim@medbo.de (M.A.); franziska.weber@medbo.de (F.C.W.); berthold.langguth@medbo.de (B.L.); martin.schecklmann@medbo.de (M.S.)

**Keywords:** depression, bipolar disorder, rtms, repetitive transcranial magnetic stimulation, non-invasive brain stimulation, neurostimulation

## Abstract

Repetitive transcranial magnetic stimulation (rTMS) is effective in the treatment of depression. However, for the subset of patients with bipolar disorder, less data is available and overall strength of evidence is weaker than for its use in unipolar depression. A cohort of 505 patients (of which 46 had a diagnosis of bipolar disorder) with depression who were treated with rTMS were analyzed retrospectively with regards to their response to several weeks of treatment. Hamilton Depression Rating Scale (HDRS) was assessed as main outcome. Unipolar and bipolar patients with depression did not differ significantly in baseline demographic variables or severity of depression. Both groups did not differ significantly in their response to treatment as indicated by absolute and relative changes in the HDRS and response and remission rates. On HDRS subitem-analysis, bipolar patients showed superior amelioration of the symptom “paranoid symptoms” in a statistically significant manner. In conclusion, depressed patients with a diagnosis of bipolar disorder benefit from rTMS in a similar fashion as patients with unipolar depression in a naturalistic setting. rTMS might be more effective in reducing paranoia in bipolar than in unipolar patients.

## 1. Introduction

Manic-depressive illness, also known as bipolar disorder, is a multifaceted psychiatric illness of significant prevalence, morbidity and mortality associated with markedly reduced quality of life and functionality, suicidality and premature death and high socioeconomic burden [1]. Bipolar depression also conveys a larger risk of psychotic symptoms than unipolar depression [2].

The management of the disorder has traditionally included pharmacological agents as well as psychological therapies [3,4] with psychotic symptoms usually requiring pharmacotherapy or electroconvulsive therapy (ECT) [2]. However, some patients do show little to no response to such treatment options or do not comply due to side effects [4] which in turn has led to an increased interest in alternative treatments such as neurostimulation.

ECT has been used for decades for the most severe forms of uni- and bipolar depression, however its comparatively invasive nature and proposed side effects on cognition and memory make it an unfavorable choice for many patients. Repetitive transcranial magnetic stimulation (rTMS) as one of the non-invasive brain stimulation (NIBS) methods has gained increasing attraction in recent years due to its easy application without the necessity of anesthesia and possibly less side effects on memory and without the side effects of anesthesia when directly compared to ECT [5,6].

rTMS as a treatment modality is noninvasive and it exerts its effects through the induction of an electromagnetic field through a magnetic coil directed over a patient’s scalp, where it induces an electrical current in the underlying are of the cortex yielding neuronal depolarization [7]. rTMS has been utilized in the treatment and management of an expanding number of psychiatric conditions given its ability to modulate the activity of certain neural circuits in a selective topographic manner. As a treatment modality, rTMS has been studied and applied with varying success in the treatment of a range of neuropsychiatric diagnoses including but not limited to affective disorders, positive and negative symptoms of schizophrenia, tinnitus or chronic pain [8,9]. Since the first US Food and Drug Administration (FDA) approval in 2008 for the treatment of major depressive disorder for the Neuronetics Neurostar System, various systems have been approved for the treatment of major depressive disorder [10].

Studies that looked at the utilization of rTMS in the therapy of bipolar disorder have mainly looked at its effects during the depression phase of the disorder, but it is worth noting that it has also been used to treat mania [4].

However, due to the rarer nature of the condition there is a lack of data on rTMS treatment for bipolar depression in the literature when compared with the number of published studies on unipolar depression and superiority over sham seems less clear than for unipolar depression, weakening the evidence base for its application in these patients [4,11,12,13,14]. Nguyen et al. presented a meta-analysis of 14 studies concluding that active rTMS is associated with a higher response rate than sham, however the authors stressed low participant number (the largest studies including only 59 patients and half of included studies including less than 10 patients) and heterogeneity of protocols as limitations [14].

Therefore, we decided to examine the effectiveness of rTMS in the subset of patients with bipolar depression in a large sample when directly compared to that in unipolar depressed patients in a naturalistic setting via retrospective analysis. Our hypothesis was that rTMS would produce beneficial effects in bipolar depression and that they would be comparable to the outcomes seen in patients with unipolar depression.

## 2. Materials and Methods

A large cohort of patients with depression who were treated with rTMS at the Center for Neuromodulation at the Department of Psychiatry and Psychotherapy of University of Regensburg (Germany) between 2002 and 2020 were analyzed retrospectively. Patients gave written informed consent to treatment. The retrospective analysis of clinical data was approved by the local ethics committee of the University of Regensburg (20-2117-104). The inclusion criteria were: naive to rTMS (only the patient’s first treatment with rTMS was considered), diagnosis of depression according to ICD-10 of F31–F33, a completed Hamilton depression rating scale (HDRS) at beginning and at the end of the rTMS treatment and absence of a serious somatic illness [15]. Both in- and outpatients were included. Based on these criteria, a sample of 505 patients could be selected for this analysis.

We have reported previously on patients of this cohort with regards to rTMS outcomes, however with a then different and/or smaller samples and different objectives [16,17,18,19,20].

Of these patients, 9.1% (46 out of 505) were diagnosed with a bipolar disorder. In the sample of the patients with unipolar depression, 29.7% (*n* = 150) suffered from the first depressive episode and 61.2% (*n* = 309) had a recurrent depressive disorder. Both groups with unipolar depression were summarized in one group for this analysis as the aim of the study was the effectiveness of rTMS in bipolar depression. The descriptive sample characteristics can be seen in Table 1. Different study protocols were used—most were treated with high-frequency protocols over the left DLPFC (*n* = 454). Three patients were stimulated on the right DLPFC, 16 on the medial prefrontal cortex and 32 were stimulated on both the left and right DLPFC in consecutive order. 

All data were analyzed using SPSS (International Business Machines Corporation, Armonk, NY, USA; Version 24.0.0.0). The significance level was set at *p* < 0.05. For group comparisons, we used Student *t*-tests or chi-square-tests depending on the scales of measurement. Response was defined as a decrease of the HDRS total score of at least 50% from pre to post rTMS and remission as a HDRS score at end of treatment below 11 points. As measures for effect size we used Cohen’s d for the relative and absolute change in the HDRS total score as indicated by G*Power 3.1.9.2 [21].

## 3. Results

**Figure 1:**
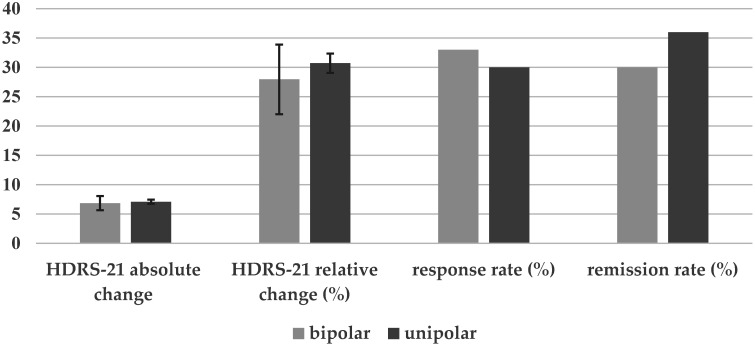
Absolute HDRS-21 change (in amount) and relative HDRS-21 change, response rate and remission rate (in percentages) for bipolar and unipolar depressed patients.

**Figure 2:**
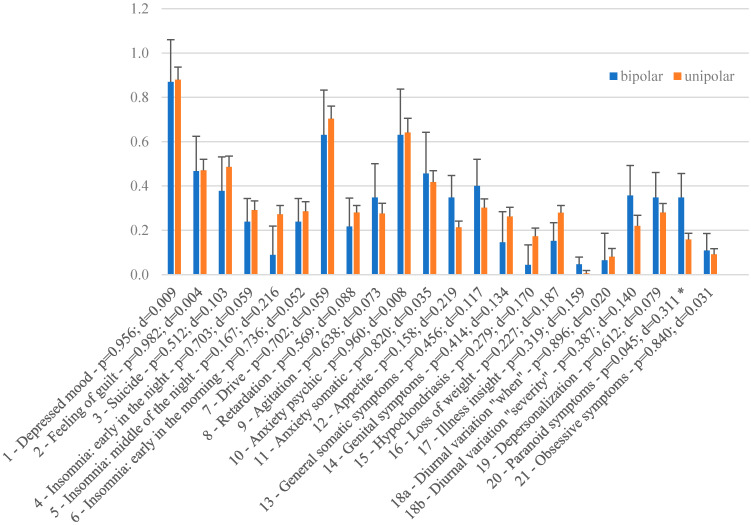
Absolute change in HDRS-21 subitems for bipolar and unipolar depressed patients. Asterisk (*) denotes items for which *p* < 0.05.

Groups did not differ with respect to demographic variables, depression severity or treatment parameters (Table 1). Table 2 indicates the frequency of taken medication. In a significant manner, bipolar patients were prescribed mood stabilizers more often and selective serotonine-norepinephrine reuptake inhibitors (SNRIs) less often. Overall, patients showed an amelioration of symptoms as indicated by a significant decrease of the HDRS-21 sum score (T = 20.582; df = 504; *p* < 0.001; d = 0.916). Both groups did not differ significantly with respect to treatment efficacy as indicated by the absolute and relative change of the HDRS-21 sum score. The effect sizes were negligible. In addition, response and remission rate based on the HDRS-21 sum score were not significantly different (Figure 1). No differences were found as to which subitems of the HDRS were altered after treatment when comparing unipolar and bipolar patients with the exception of the item “paranoid symptoms” (Figure 2). For this item, patients with bipolar depression showed significantly more reduction after rTMS treatment than their unipolar counterparts (*p* = 0.045).

## 4. Discussion

Our analysis, which included a large sample of 505 in- and outpatients, revealed a marked and similar decrease in depression symptoms in both unipolar as well as bipolar depression under rTMS as measured by the HRDS. Baseline depression score and demographic characteristics were not significantly different, indicating adequate comparability of the groups.

A prevalence of 9.1% bipolar patients in our large sample corresponds to the lower prevalence of the illness when compared to unipolar patients [1], highlighting one of the reasons why fewer studies exist in this population.

Increased use of mood stabilizers in the group with bipolar depression was anticipated due to their common use in this patient population. Previous work has shown that intake of these medications aswell as that of lithium is not associated with inferior treatment outcomes in the naturalistic setting, providing evidence against the theoretical concern that drugs with an anticonvulsive mechanism of action might hamper with rTMS effects [20]. The less widespread use of SNRIs in the group of bipolar patients might be associated with concerns of increased risk of inducing mania.

Apart from the lower number of controlled studies in bipolar patients, recent studies have also failed to show superiority of certain rTMS protocols over sham in this population [13,22] while another rather large study could demonstrate superiority, but for the rather specialized and rarely used in everyday practice protocol of deep rTMS [23]. The meta-analysis by Nguyen et al. supports superiority over sham in the light of limitation by low participant numbers, but makes no claim about the direct comparison between uni- and bipolar patients [14]. With this paper, we add to the evidence that for the comparatively novel method of rTMS, there is similar equal effectiveness in both types of depression when compared with each other directly in a naturalistic, retrospective setting.

The findings of equal treatment outcome make sense as the symptomatology and neurobiology of unipolar and bipolar depression share numerous similarities and may encourage clinicians to offer rTMS treatment to their patients with bipolar depression [24]. 

Sub-item analysis of the HDRS in our study also revealed no significant difference as to which depressive symptoms were altered by rTMS with the exception of paranoid symptoms, which were alleviated more in a statistically significant manner in the patients with bipolar depression. This finding must however be interpreted cautiously, as running the analysis on all 21 sub-items increases the statistical chance of identifying at least on significant outcome. On the other hand, identifying significance on this special item might yield clues to underlying mechanisms of rTMS on the conditions in question. rTMS of the DLPFC has been used to treat negative symptoms of schizophrenia, which show similarities to depression [8]. However, when applied for reduction of productive psychotic symptoms, other cortical areas are usually targeted, such as the temporoparietal cortex [8]. Therefore, and considering equal relative intake of antipsychotics in the groups (Table 2), we suspect the reduction of paranoid delusion in our depressed patients to be a secondary effect of depression alleviation. The difference between the groups might indicate differences in the underlying neurobiology of paranoia in unipolar and bipolar depression with better responsiveness to rTMS treatment in the latter phenotype. Little data on this matter exists, probably due to ethical and practical challenges in conducting studies on patients with psychotic features [25] but some evidence points towards psychotic depression being associated with abnormal functional connectivity [25] which in principal can be modulated by rTMS [26]. However, conclusions on potential mechanisms on the matter are premature and these findings should be replicated and then investigated further with respect to underlying mechanisms.

A weakness of our study is its retrospective nature and lack of a prospective, controlled matched comparison between the uni- and bipolar groups and the use of unipolar depressed patients as a control group instead of sham treatment. However, a major strength is the large patient number and the realistic sample of seriously ill- and outpatients at a tertiary hospital with numerous pharmaceutical agents as co-therapy. As rTMS is currently very rarely used as a first-line treatment [7,11] these patients represent a very realistic sample of those who would receive rTMS as a treatment.

A limiting factor is that our results apply only to the rTMS protocols used as outlined in the Methods section with high-frequency rTMS over the left dorsolateral prefrontal cortex being used, with heterogeneity of treatment protocols in the literature being one of the reasons for the limited evidence base on treatment of bipolar depression [14].

## 5. Conclusions

rTMS was as efficient in the treatment of bipolar depression as in that of unipolar depression in a large naturalistic sample with equal baseline characteristics of the two partient groups. Further research is warranted to demonstrate superiority of rTMS over sham in the treatment of bipolar depression and to evaluate differences in efficacy of various rTMS treatment protocols. rTMS might be more effective in reducing paranoia in bipolar than in unipolar patients. The latter finding remains to be replicated and if valid, warrants further investigation.

## Figures and Tables

**Table 1:** Characteristics of patients with depression.

	Bipolar (*n* = 46)	Unipolar (*n* = 459)	Statistics for Group Contrasts
age (years)	48 ± 13	47 ± 13	T = 0.726; df = 503; *p* = 0.468
sex (female/male)	25/21	248/211	χ^2^ = 0.002; df = 1; *p* = 0.967
resting motor threshold	44 ± 12	43 ± 9	T = 0.922; df = 500; *p* = 0.357
stimulation intensity	46 ± 9	45 ± 8	T = 0.617; df = 503; *p* = 0.538
number of pulses per session	1935 ± 370	1876 ± 407	T = 0.938; df = 503; *p* = 0.349
number of sessions per patient/treatment	19 ± 6	18 ± 6	T = 0.874; df = 503; *p* = 0.383
HDRS-21 baseline	22 ± 8	21 ± 7	T = 0.196; df = 503; *p* = 0.845
HDRS-21 absolute change (from pre to post treatment)	7 ± 8	7 ± 8	T = 0.198; df = 503; *p* = 0.843; d = 0.030
HDRS-21 relative change (%; from pre to post treatment)	28 ± 40	31 ± 36	T = 0.493; df = 503; *p* = 0.622; d = 0.072
response rate [yes/no] (relative frequency of responders)	15/31 (33%)	139/320 (30%)	χ^2^ = 0.107; df = 1; *p* = 0.744
remission rate (yes/no)	14/32 (30%)	167/292 (36%)	χ^2^ = 0.643; df = 1; *p* = 0.422

**Table 2:** Medication intake.

	Bipolar (*n* = 39)	Unipolar (*n* = 395)	Statistics for Group Contrasts (df = 1)
selective serotonin reuptake inhibitors	14	166	χ^2^ = 0.549; *p* = 0.459
serotonin-norepinephrine reuptake inhibitors	14	208	χ^2^ = 3.991; *p* = 0.046
tricyclic antidepressants	11	115	χ^2^ = 0.014; *p* = 0.905
tetracyclic antidepressants	0	2	χ^2^ = 0.198; *p* = 0.656
monoamine oxidase inhibitors	2	11	χ^2^ = 0.671; *p* = 0.413
benzodiazepines	13	124	χ^2^ = 0.062; *p* = 0.804
z-drugs	4	43	χ^2^ = 0.015; *p* = 0.904
mood stabilizers	36	117	χ^2^ = 61.110; *p* < 0.001
antipsychotics	29	247	χ^2^ = 2.145; *p* = 0.143
other antidepressants	14	166	χ^2^ = 0.549; *p* = 0.459

The number in each cell indicates how many patients of the respective diagnostic group were taking medications of the indicated classification. Please notice that for 71 out of 505 patients no valid medication information was available.

## Data Availability

The data is not contained in a publicly accessible data repository.
